# Strategy for Scanning Peptide-Coding Circular RNAs in Colorectal Cancer Based on Bioinformatics Analysis and Experimental Assays

**DOI:** 10.3389/fcell.2021.815895

**Published:** 2022-02-25

**Authors:** Zhanghan Chen, Zhipeng Qi, Dongli He, Jingyi Liu, Enpan Xu, Bing Li, Shilun Cai, Di Sun, Yirong Cheng, Qiang Shi, Yunshi Zhong

**Affiliations:** ^1^ Endoscopy Center, Zhongshan Hospital of Fudan University, Shanghai, China; ^2^ Endoscopy Research Institute of Fudan University, Shanghai, China; ^3^ Department of Internal Medicine of Xuhui Hospital, Affiliated Zhongshan Hospital, Fudan University, Shanghai, China

**Keywords:** colorectal cancer, circRNAs, short peptides, LC-MS/MS, bioinformatic analysis

## Abstract

**Background:** Colorectal cancer (CRC) is the third most common cause of cancer deaths worldwide. Numerous studies have reported that circular RNAs (circRNAs) have important functions in CRC. It was first thought that circRNAs were non-coding RNA; however, more recently they were discovered to encode peptides and play a pivotal role in cancer development and progression. It was shown that most circRNAs possess coding potential; however, not all of them can truly encode peptides. Therefore, a practical strategy to scan for coding circRNAs is needed.

**Method:** Sequence analyses included open reading frame (ORF) prediction, coding peptide prediction, and the identification of unique sequences. Then, experimental assays were used to verify the coded peptides, liquid chromatography-tandem mass spectrometry (LC-MS/MS) was introduced to detect sequences of circRNAs with coding potential, and Western blot was used to identify the encoded peptides. Finally, the functions of the circRNAs were primarily explored.

**Result:** An efficient strategy for searching circRNAs with coding potential was created. We verified this schedule using public databases and LC-MS/MS, then two of these circRNAs were selected for further verification. We used commercial antibodies that can also identify the predicted peptides to test the coded peptides. The functions of the circRNAs were explored primarily, and the results showed that they were mainly involved in the promotion of proliferation and invasion ability.

**Discussion:** We have constructed an efficient strategy of scanning circRNAs with coding potential. Our strategy helped to provide a more convenient pathway for identifying circRNA-derived peptides, which can be a potential therapeutic target or a diagnostic biomarker.

## Introduction

Colorectal cancer (CRC) is now ranked third in terms of incidence and second in terms of mortality of all cancers worldwide (Sung et al., 2021). Even with the popularization of endoscopic surgery and the improvement of CRC treatment, efficient and potent biomarkers for early diagnosis and therapeutic targets are still needed (Siegel et al., 2020; Sung et al., 2021). Circular RNAs (circRNAs), a group of RNAs that possess a cyclic structure, were once believed to be non-coding and were reported to play a pivotal role in tumorigenesis and tumor development (Artemaki et al., 2020). Mechanistically, the circRNAs can act as miRNA sponges and regulate downstream targets. Previous research found that circCAMSAP1 regulated by ESRP1 could sponge miR-328-5p and then target E2F1 to mediate CRC progression (Zhou et al., 2020), and circHIPK3 could promote CRC proliferation and metastasis through the c-Myb/circHIPK3/miR-7 axis (Zeng et al., 2018). Moreover, circRNA can also interact with proteins and function as a tumor suppressor or promoter (Long et al., 2021). Researchers found that circPTK2 could promote the epithelial–mesenchymal transition (EMT) of CRC cells by interacting with the Ser38, Ser55, and Ser82 sites of the vimentin protein (Yang et al., 2020). Another circRNA, circ_cse1l, can downregulate the expression of *PCNA* and inhibit the proliferation of CRC cells by binding to eIF4A3 (Xu et al., 2020). It was not until recent years that circRNAs were discovered to have coding potential. Legnini et al. have identified a protein encoded by circ-ZNF609 in myogenesis (Legnini et al., 2017; Pamudurti et al., 2017). Moreover, researchers have determined the translatomes of 80 human hearts and identified undetected microproteins, which also included peptides encoded by circRNAs (van Heesch et al., 2019). Moreover, Zhang and his colleagues found several circRNAs that could encode peptides that functioned significantly in glioma (Yang et al., 2018; Zhang et al., 2018; Xia et al., 2019; Gao et al., 2021; Wu et al., 2021a). Furthermore, the coding potential of circRNAs can also be observed in liver cancer (Liang et al., 2019; Li et al., 2021), gastric cancer (Jiang et al., 2021), and colon cancer (Zheng et al., 2019; Pan et al., 2020). Therefore, exploring the coding potential and functions of circRNAs is important. In this study, we developed an effective schedule to scan circRNAs that possessed coding potential in CRC and found two circRNAs that were most likely to encode peptides. Preliminarily, we verified the existence of coded peptides and the functions of their derived circRNAs.

## Materials and Methods

### Selection and Profiling of RNA Datasets

All eligible microarray datasets available up to December 2020 were downloaded from GEO (http://www.ncbi.nlm.nih.gov/gds/). The following search words were used: (circular RNA or circRNA) and (CRC or colorectal cancer). The filters were as follows: 1) at least five pairs of normal and cancer tissues, 2) no metastasis, 3) human solid tissues, and 4) arrays should have differentially expressed circRNAs between normal and cancer tissue after differential analysis. Two datasets were finally included in this study: GSE126095 and GSE142837. The R software was used to calibrate, standardize, and log_2_ transform the downloaded files.

### Open Reading Frame Prediction of circRNAs

The original sequences of circRNAs were downloaded from the circBase (http://www.circbase.org/). Then the open reading frames (ORFs) were predicted by using the getorf database (http://emboss.bioinformatics.nl/cgi-bin/emboss/getorf) with the sequence set as “circular.” After infinite ORFs were excluded (Abe et al., 2015; Mo et al., 2019), the amino acid (a.a.) sequences that spanned the junction sites of the circRNAs were selected.

### Identification and Evaluation of the Coding Potential in Differentially Expressed circRNAs

**FIGURE 1:**
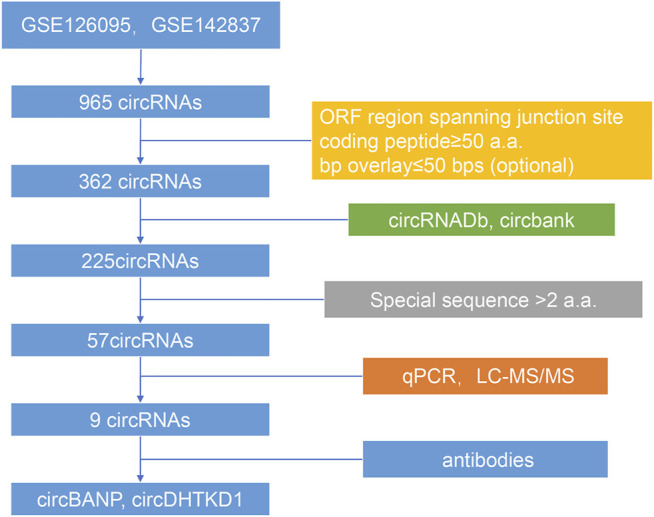
Schedule of scanning potential coding circRNAs in CRC.

The batch effects between the datasets GSE126095 and GSE142837 were normalized by the sva package. Differential analysis was performed using the Limma package. The differentially expressed circRNAs met the standards: |log_2_FC| > 1 and adjust *p*-value <0.05. The coding potential of differentially expressed circRNAs was predicted by the following rules: 1) predicted ORF regions spanned the junction sites of circRNAs, 2) the predicted coded peptides had more than 50 a.a., and 3)the bases overlay less than 50 bps, which could make the peptides easier to identify. The databases circRNADb and circbank were introduced to evaluate the overall coding potential of differentially expressed circRNAs. Finally, the circRNA-derived peptides should have a unique sequence with more than two a.a. The flowchart is shown in Figure 1.

### Cell Culture

The CRC cells HCT116, SW480, SW620, Lovo, HT29, DLD-1, and CACO2 and normal control cells NCM460 were all cultured in Dulbecco's modified essential medium (DMEM) with 10% fetal bovine serum (FBS). Cells were obtained from the Chinese Academy of Sciences (Shanghai, China) and Chuan Qiu Biotechnology (Shanghai, China).

### RNA Extraction and Real-Time Quantitative Reverse Transcription PCR Analysis

The TRIzol Reagent (Invitrogen, United States; 15596-026) (1 ml per well) was used to lyse cells, then the RNAs were extracted using RNAsimple Kit (TIANGEN, China; DP419). The concentration of RNAs was tested by DS-11 spectrophotometer (DeNovix, United States). RNA samples (2 μg) were mixed with PrimeScript™ RT reagent Kit with gDNA Eraser (TaKaRa, Tokyo, Japan; RR047A) and then applied to the ProFlex™ PCR system (ABI, United States) to obtain cDNAs. The cDNA samples were mixed with SYBR Premix Ex Taq II (TaKaRa, Tokyo, Japan; RR820A) and quantified by using ABI QuantStudio 5 (ABI, United States); the reaction was initiated at 95°C for 1 min, then at 95°C for 5 s, and 60°C 30 s for a total of 40 cycles. The primers are listed in Supplementary Table S1. Glyceraldehyde 3-phosphate dehydrogenase (GAPDH) was set as the internal reference. The 2^−ΔΔCt^ method was used to process the data.

### Western Blot Analysis

Radioimmunoprecipitation assay (RIPA) lysis buffer (Beyotime, China; P0013B) and protease and phosphatase inhibitor cocktail for general use (Beyotime, China; P1045) were used to obtain protein samples. Protein samples were denatured at 100°C for 10 min. Electrophoresis was started at 80 V to concentrate the protein and 120 V to separate the protein, then the transfer was conducted for 1 h under 300 mA. After blocking for 1 h with 5% non-fat milk (BD Biosciences, United States; 232100), the membranes were incubated overnight with primary antibodies BANP (ABclonal, China; A7595) and DHTKD1 (Santa Cruz Biotechnology, Inc., United States; sc-398620) at 4°C. After washing away the primary antibody with Tris-buffered saline Tween-20 (TBST), the secondary antibody (Abmart, China; M21003) was incubated for 1 h at room temperature. The bands were detected in automatic chemiluminescence and fluorescence analysis system (Tanon, China) by adding enhanced chemiluminescent reagent (NCM Biotech, China; P2200).

### Liquid Chromatography-Tandem Mass Spectrometry Analysis

To identify the unique a.a. sequence of circRNAs encoded peptides, the total protein was subjected to liquid chromatography-tandem mass spectrometry (LC-MS/MS) sequencing and data analysis by Oebiotech Co., Ltd. (Shanghai, China). In brief, enzymolysis was performed using 0.02 μg/μl trypsin, and then the peptides were desalinated. The sample was analyzed using a Nano-HPLC liquid phase system EASY-NLC1200 and a Q-Exactive mass spectrometer. The acquired data were analyzed using ProteomeDiscover (V2.4, Thermo, United States) software.

### Sanger Sequencing

The cDNA from DLD-1 were amplified by PCR using a primer specifically targeting hsa_circ_0000725 and hsa_circ_0008826 and 2×Hieff ®PCR Master Mix (with dye) (Yeasen, China; 10102ES03). To confirm the junction sites, Sanger sequencing was conducted by using the primers listed in Supplementary Table S1.

### Construction and Transfection of siRNA

To silence the circRNAs, siRNAs were synthesized by RIBOBIO (Guangzhou, China). Lipofectamine™ RNAiMAX Transfection Reagent (Invitrogen, United States; 13778150) was used to transfect the siRNAs, and the efficiency was evaluated by qPCR.

### EdU and CCK-8 Cell Proliferation Assays

Cells (2,000 per well) were seeded in 96-well plates. EdU (RIBOBIO, China; C10310-3) and CCK-8 (BBI, China; E606335) reagents were used to estimate cell proliferative ability following the manufacture instructions. The images of EdU were analyzed by ImageJ, and the GraphPad Prism 7.0 software (GraphPad Software Inc., La Jolla, CA, United States) was used to analyze CCK-8 results.

### Colony Formation Assay

Cells (2,000 per well) were seeded in each well of a six-well plate. After 14 days of culture, the cells were fixed by 4% paraformaldehyde and stained by crystal violet. The images were analyzed by ImageJ.

### Invasion Assay

To evaluate cell invasion ability, each upper chamber (Biofil, China; TCS003024) with a 1:7 concentration of Matrigel membrane (BD Biosciences, United States; 356234) was seeded with 50,000 cells with the serum-free culture medium. The lower chambers were put into complete culture medium. After 72 h, the cells in the chambers were fixed by 4% paraformaldehyde and stained by crystal violet. Then, the upper chamber cells were cleared. The images were analyzed by ImageJ.

### Flow Cytometry of Cell Cycle and Apoptosis

In the cell cycle test, the cells were collected with cold phosphate-buffered saline (PBS) and fixed in 70% cold ethanol at −20°C overnight. After staining with propidine iodide (BD Biosciences, United States) for 30 min, the cells were loaded into a flow cytometer (BD Biosciences, United States), then 20,000–30,000 cells per tube were collected, and the results were analyzed by FlowJo (Version 10, United States). In the apoptosis test, the cells were suspended in Annexin-V binding buffer and then dyed using Annexin V combined fluorescein isothiocyanate (BD Biosciences, United States) and propidine iodide (BD Biosciences, United States) simultaneously in the dark for 15 min. The cells were loaded into a flow cytometer (BD Biosciences, United States), then 20,000–30,000 cells per tube were collected, and the results were analyzed by FlowJo (Version 10, United States).

### Statistical Analyses

The Student's *t*-test was used to evaluate the difference. A two-sided *p*-value <0.05 was considered statistically significant. Statistical analysis and graphs were constructed using GraphPad Prism 7.0 software (GraphPad Software Inc., La Jolla, CA, United States).

## Results

### Evaluation of the Coding Potential of Differentially Expressed circRNAs in CRC

**FIGURE 2:**
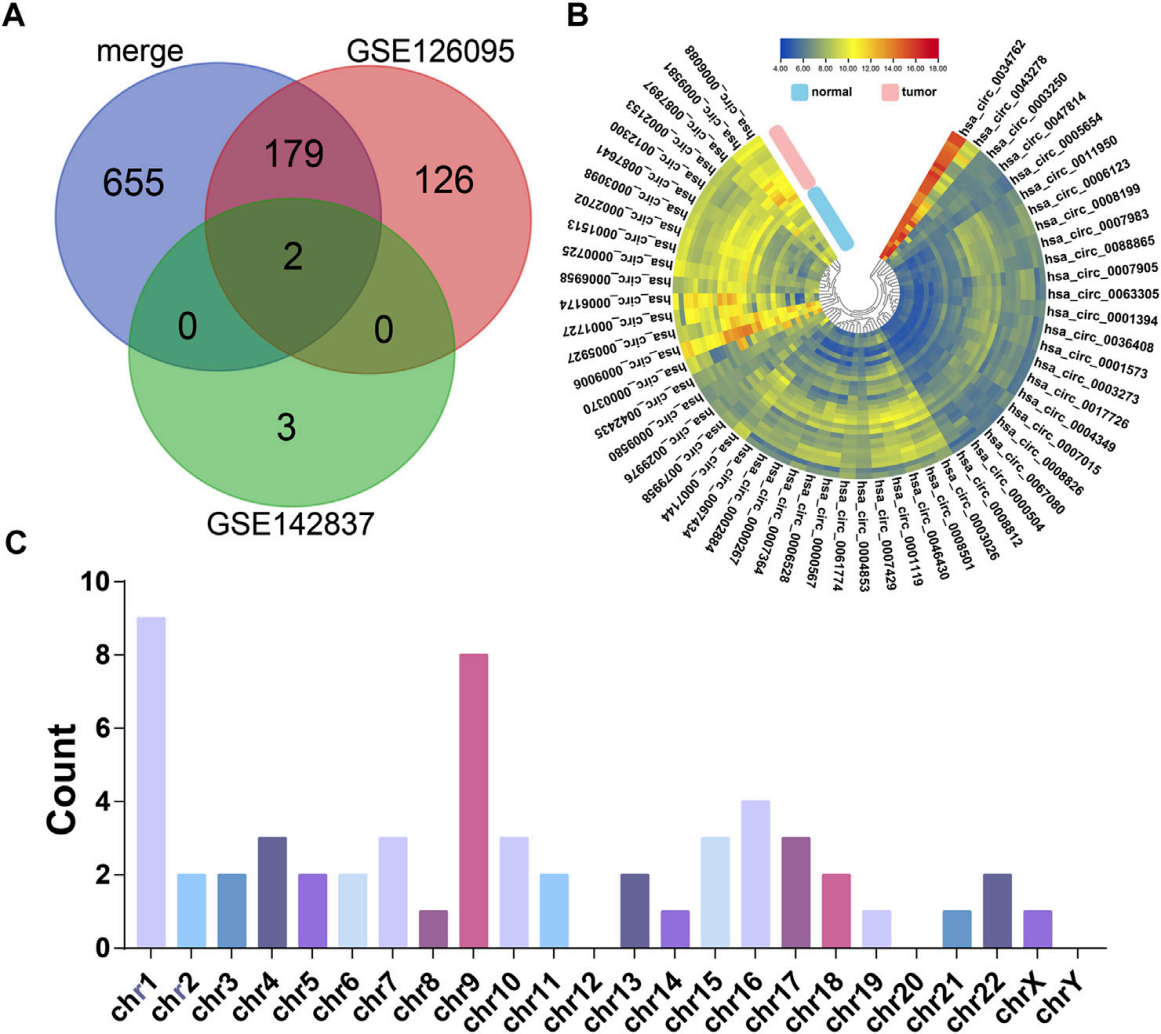
The candidates of coding potential circRNAs. **(A)** Differential analysis of GSE126095 and GSE142837. **(B)** Heat map of the 57 circRNAs with coding potential in GSE126095 and GSE142837 after filtering by sequence and databases. **(C)** Chromosome location summary of 57 circRNAs with coding potential.

According to our strategy (Figure 1), we scanned the circRNAs with coding potential in CRC. First, to extend the candidate list, we compared the normal and CRC samples in the microarray datasets both together and separately. After deduplicating, the differential analysis found 965 differentially expressed circRNAs, including 836 circRNAs from the merge of GSE126095 and GSE142837 microarray datasets, 307 circRNAs from the GSE126095, and 5 circRNAs from the GSE142837 (Figure 2A). Then we analyzed the sequences of these circRNAs; the predicted ORF should span the junction site of their circRNAs, and the predicted peptides should be over 50 a.a. so that the peptides were not too small to be detected. The circular structure of the circRNAs enabled their bps to be used more than one time. However, this repeated use can make the analysis very difficult, so we mainly focused on circRNAs that were predicted to have less than 50 bps for repeated use. This resulted in the exclusion of 603 circRNAs and left 362 circRNAs. Next, the databases circRNADb and circbank were introduced to evaluate the potential coding function, and 225 circRNAs were selected. Special sequences in the coding peptides from circRNAs were the identification codes of circRNAs themselves; we selected circRNAs that had more than two unique a.a. to ensure the quality of the following LC-MS/MS assays because only one unique a.a. can have many deviations, and the more a.a. detected the more convincing the results will be. A total of 57 circRNAs were identified (Table 1), containing 46 upregulated circRNAs and 11 downregulated circRNAs Figure 2B. Besides, most of these circRNAs were derived from chromosomes 2 and 9, which may indicate their functions (Figure 2C).

**TABLE 1:** The basic information of 57 coding potential circRNAs.

ID	Log FC	Host gene	Best transcript	Position
hsa_circ_0006174	2.96	RAD23B	NM_002874	chr9:110064315-110068928
hsa_circ_0001727	2.05	ZKSCAN1	NM_003439	chr7:99621041-99621930
hsa_circ_0009581	1.95	RERE	NM_012102	chr1:8555122-8601377
hsa_circ_0046430	1.94	FOXK2	NM_004514	chr17:80521229-80545148
hsa_circ_0002702	1.91	RUSC2	NM_001135999	chr9:35546426-35548532
hsa_circ_0000567	1.81	SETD3	NM_199123	chr14:99924615-99932150
hsa_circ_0003098	1.72	BANP	NM_001173542	chr16:88037900-88071617
hsa_circ_0007429	1.71	RREB1	NM_001003699	chr6:7181356-7189555
hsa_circ_0001119	1.69	NDUFA10	NM_004544	chr2:240929490-240954277
hsa_circ_0006528	1.61	PRELID2	NM_138492	chr5:145197456-145205763
hsa_circ_0003026	1.56	USP10	NM_005153	chr16:84773914-84779279
hsa_circ_0063305	1.55	CSNK1E	NM_152221	chr22:38698865-38699253
hsa_circ_0087641	1.49	CDC14B	NM_033331	chr9:99284787-99327765
hsa_circ_0011950	1.48	HIVEP3	NM_024503	chr1:42041214-42050989
hsa_circ_0007144	1.45	PTPRM	NM_001105244	chr18:8076452-8143777
hsa_circ_0006123	1.43	KCNH1	NM_172362	chr1:211092981-211093411
hsa_circ_0001394	1.41	TBC1D14	NM_001113361	chr4:6925099-6925838
hsa_circ_0000725	1.40	BANP	NM_001173539	chr16:88008653-88052273
hsa_circ_0002884	1.33	PICALM	NM_007166	chr11:85692171-85707972
hsa_circ_0012300	1.31	PIK3R3	NM_003629	chr1:46521466-46546422
hsa_circ_0007983	1.31	GABPB1	NM_005254	chr15:50592985-50596330
hsa_circ_0067434	1.31	RYK	NM_001005861	chr3:133894452-133901915
hsa_circ_0007905	1.30	STX6	NM_005819	chr1:180953812-180962561
hsa_circ_0007364	1.28	PTP4A2	NM_080391	chr1:32381495-32385259
hsa_circ_0001573	1.25	RREB1	NM_001168344	chr6:7176887-7189555
hsa_circ_0007015	1.24	PTPN14	NM_005401	chr1:214625147-214638300
hsa_circ_0017726	1.23	DHTKD1	NM_018706	chr10:12123470-12139995
hsa_circ_0000267	1.22	FAM53B	NM_014661	chr10:126370175-126370948
hsa_circ_0004349	1.21	RAC1	NM_018890	chr7:6426842-6431672
hsa_circ_0061774	1.19	BACE2	NM_012105	chr21:42598192-42629253
hsa_circ_0006958	1.19	ACSF3	NM_001243279	chr16:89164998-89169167
hsa_circ_0008199	1.18	ATXN10	NM_013236	chr22:46085591-46136418
hsa_circ_0001513	1.15	LNPEP	NM_005575	chr5:96314841-96322374
hsa_circ_0008812	1.14	RAD23B	NM_002874	chr9:110062421-110074018
hsa_circ_0079958	1.13	HECW1	NM_015052	chr7:43508518-43540921
hsa_circ_0008501	1.12	RERE	NM_012102	chr1:8601272-8674745
hsa_circ_0088865	1.09	SPTAN1	NM_001130438	chr9:131365821-131367756
hsa_circ_0004853	1.07	CD97	NM_078481	chr19:14513408-14515374
hsa_circ_0067080	1.06	ITGB5	NM_002213	chr3:124560229-124592378
hsa_circ_0008826	1.06	DHTKD1	NM_018706	chr10:12136071-12162266
hsa_circ_0009006	1.05	LDB2	NM_001130834	chr4:16587544-16597498
hsa_circ_0006088	1.05	SPTAN1	NM_001130438	chr9:131362358-131367756
hsa_circ_0002153	1.03	MID2	NM_012216	chrX:107083899-107097934
hsa_circ_0000504	1.02	TUBGCP3	NM_006322	chr13:113170753-113181798
hsa_circ_0003273	1.02	TRIP12	NM_004238	chr2:230723487-230744844
hsa_circ_0036408	1.00	ETFA	NM_000126	chr15:76580186-76588078
hsa_circ_0005654	−1.04	PRDM5	NM_018699	chr4:121675707-121732604
hsa_circ_0087897	−1.15	C9orf5	NM_032012	chr9:111795586-111870850
hsa_circ_0047814	−1.23	ZNF532	NM_018181	chr18:56585502-56587865
hsa_circ_0005927	−1.25	VDAC3	NM_001135694	chr8:42259305-42260979
hsa_circ_0029976	−1.37	NBEA	NM_015678	chr13:35615069-35644989
hsa_circ_0003250	−1.38	MRRF	NM_138777	chr9:125042721-125054119
hsa_circ_0000370	−1.52	FLI1	NM_002017	chr11:128628009-128651918
hsa_circ_0034762	−1.57	MAPKBP1	NM_001128608	chr15:42103080-42105299
hsa_circ_0009580	−1.65	RERE	NM_012102	chr1:8555122-8568734
hsa_circ_0042435	−1.76	SPECC1	NM_001243439	chr17:20149238-20209395
hsa_circ_0043278	−5.12	TADA2A	NM_001488	chr17:35797838-35800763

### Expression of Candidate circRNAs in CRC

**FIGURE 3:**
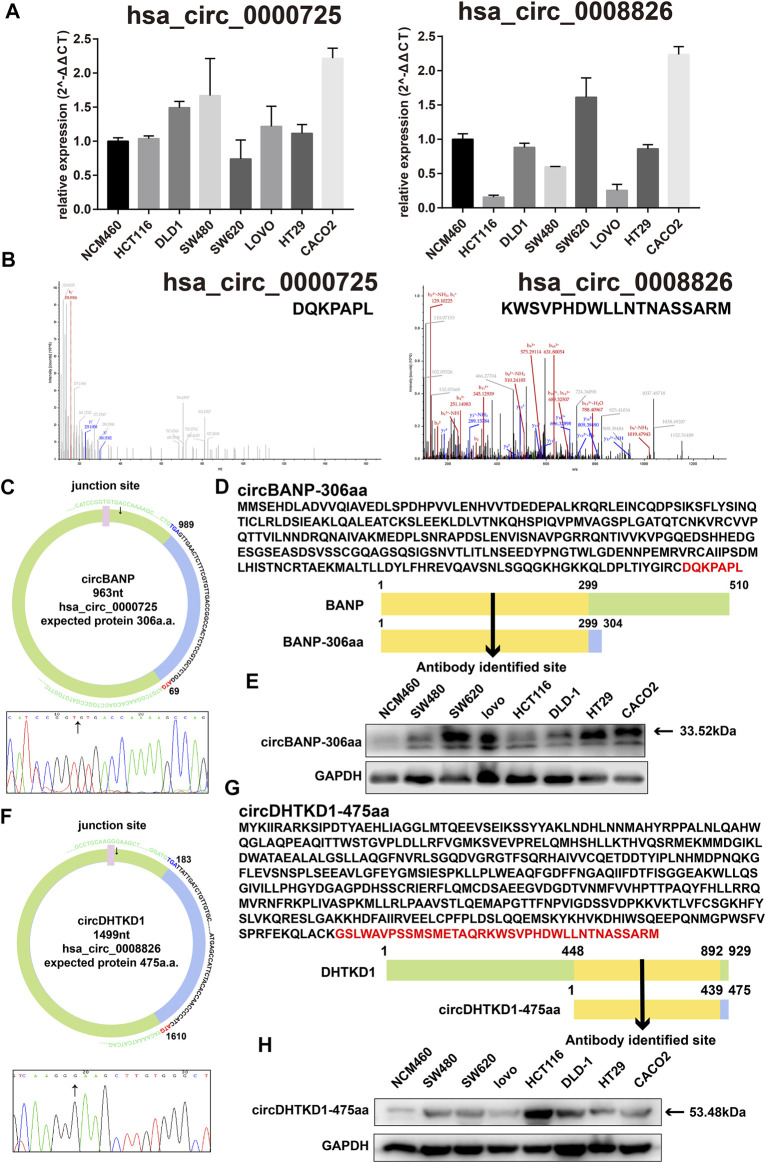
Basic information of hsa_circ_0000725 and hsa_circ_0008826. **(A)** Expression level of hsa_circ_0000725 and hsa_circ_0008826 in CRC cells and normal cells. **(B)** LC-MS/MS found the unique sequence of hsa_circ_0000725 and hsa_circ_0008826. **(C)** The structure and junction site of hsa_circ_0000725. **(D)** Sequence alignment of hsa_circ_0000725 to its host gene. **(E)** The expression of circBANP-306aa in CRC cells. **(F)** The structure and junction site of hsa_circ_0008826. **(G)** Sequence alignment of hsa_circ_0008826 to its host gene. **(H)** The expression of circDHTKD1-475aa in CRC cells.

Experimentally, we tested the basic expression levels of the 57 circRNAs in a panel of seven CRC cell lines and one normal colorectal cell line NCM460 (data available under request). From those circRNAs, hsa_circ_0000725, hsa_circ_0007429, hsa_circ_0008501, and hsa_circ_0067080 were expressed significantly higher in most CRC cells, whereas hsa_circ_0005654 was expressed significantly lower in most CRC cells, which is the same as the differential analysis. However, the expressions of hsa_circ_0006088, hsa_circ_0007364, hsa_circ_0008199, and hsa_circ_0008826 were slightly varied in different CRC cells (Figure 3A and Supplementary Figure S1A).

### Coding Potential Evaluation of Candidate circRNAs

Sequencing analysis found the unique a.a. sequence coded by the candidate circRNAs (Supplementary Table S2). We also performed LC-MS/MS analysis to identify the 57 circRNAs. Strikingly, the LC-MS/MS results found the special sequences of nine circRNAs, including hsa_circ_0000725, has_circ_0007429, hsa_circ_0008501, hsa_circ_0067080, hsa_circ_0005654, hsa_circ_0006088, hsa_circ_0007364, hsa_circ_0008199, and hsa_circ_0008826 (Figure 3B and Supplementary Figure S1B). Exploration of the circRNAs showed that the hsa_circ_0000725 contained a complete ORF with 963 nt and might encode a 306-a.a. peptide; hsa_circ_0008826 contained a 1,499 nt ORF with the potential of encoding a 475-a.a. length peptide (Figures 3C, F). The ORF structures of hsa_circ_0007429, hsa_circ_0008501, hsa_circ_0067080, hsa_circ_0005654, hsa_circ_0006088, hsa_circ_0007364, and hsa_circ_0008199 are shown in Supplementary Figure S2. The a.a. sequence encoded by hsa_circ_0000725 had a 299-a.a. area in common with its host gene BANP; therefore, a commercial BANP antibody that targeted this area (Figure 3D) was used to detect the circBANP-306aa, and the Western blot identified a band at about 33.52 kDa (Figure 3E). Also, the a.a. encoded by hsa_circ_0008826 had a 439-a.a. area in common with its host gene DHTKD1; a commercial DHTKD1 antibody that targets this area (Figure 3G) was used to detect circDHTKD1-475aa, and the Western blot identified a band at about 53.48 kDa area (Figure 3H).

### Silencing of hsa_circ_0000725 and hsa_circ_0008826 can Inhibit CRC Cell Proliferation

**FIGURE 4:**
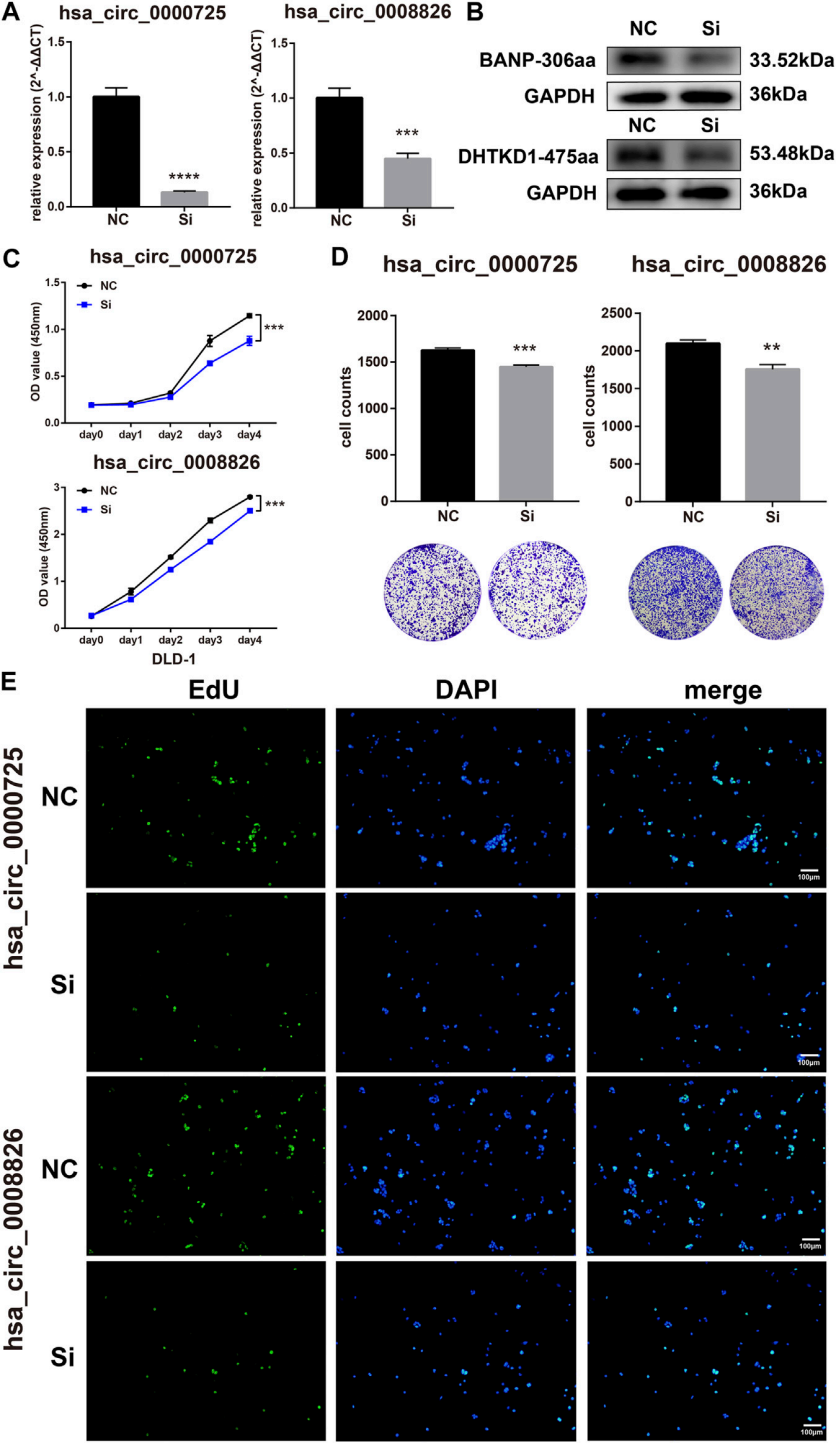
Hsa_circ_0000725 and hsa_circ_0008826 influence the proliferation ability of CRC cells. **(A,B)** efficiency evaluation of siRNAs. **(C)** CCK8 assay showed the silencing of hsa_circ_0000725 and hsa_circ_0008826 decreased the proliferation ability. **(D)** Colony formation assay showed that the silencing of hsa_circ_0000725 and hsa_circ_0008826 weakened colony formation ability. **(E)** EdU assay showed the silencing of hsa_circ_0000725 and hsa_circ_0008826 reduced the proliferation rate of CRC cells.

The function of circRNAs-derived coding peptides may significantly relate to their derived circRNA. Therefore, we preliminarily explored the function of hsa_circ_0000725 and hsa_circ_0008826; their siRNAs were constructed and transfected into DLD-1. The siRNAs could efficiently silence the hsa_circ_0000725 and hsa_circ_0008826 in DLD-1 (Figures 4A, B). The CCK8 proliferation assay showed that the silencing of hsa_circ_0000725 and hsa_circ_0008826 could significantly inhibit the proliferation ability of DLD-1(Figure 4C). The colony formation assay indicated that the silencing of hsa_circ_0000725 and hsa_circ_0008826 could significantly decrease the colony-forming ability of the DLD-1 (Figure 4D). The EdU staining also apparently showed the number of cells in a proliferation state decreased significantly when the silenced hsa_circ_0000725 and hsa_circ_0008826 were compared to the normal control in DLD-1 (Figure 4E).

### Silencing of hsa_circ_0000725 and hsa_circ_0008826 Could Also Influence the Cell Cycle, Apoptosis, and Invasion Ability of CRC Cells

**FIGURE 5:**
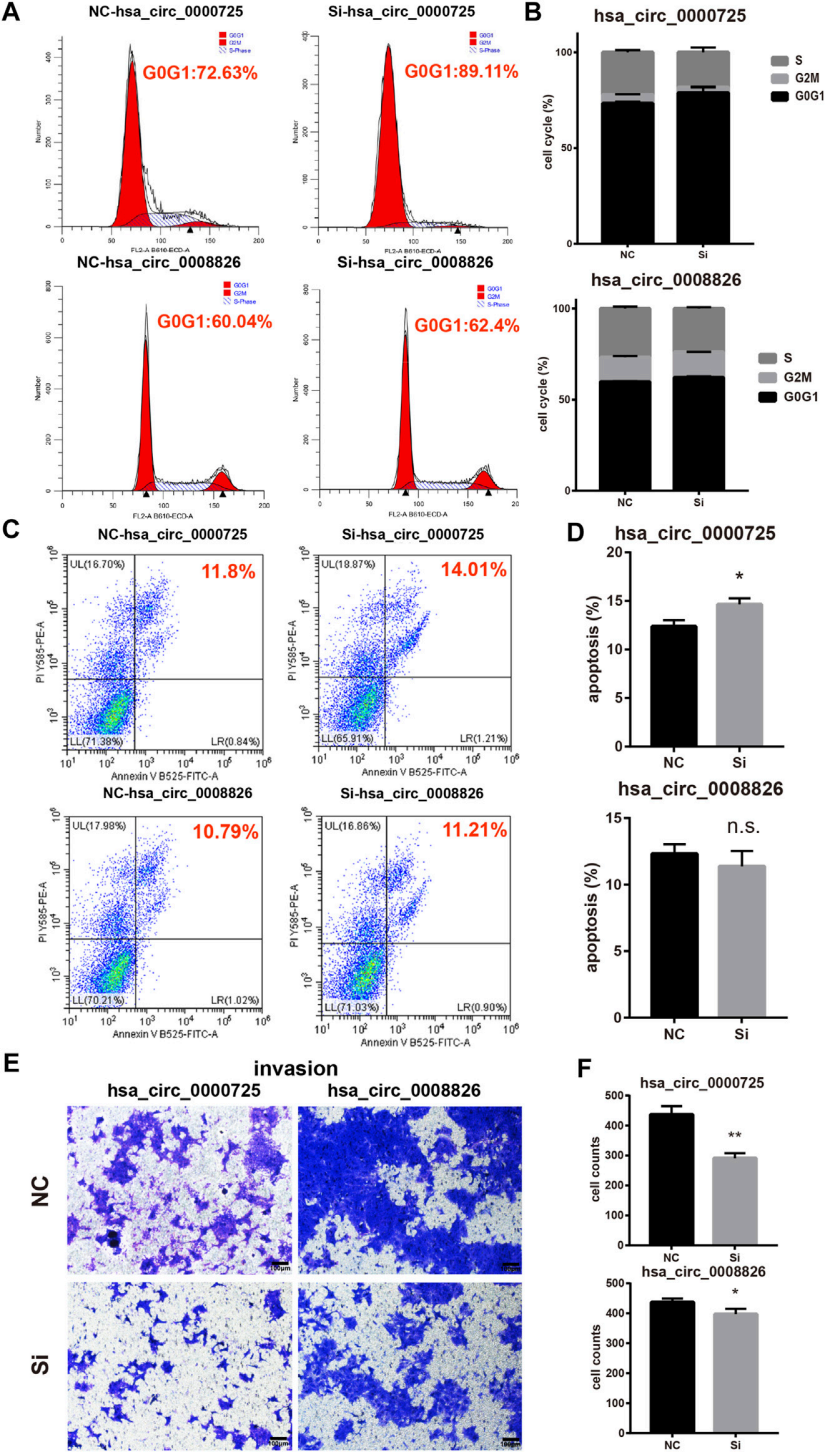
Other function assays of hsa_circ_0000725 and hsa_circ_0008826. **(A,B)** cell cycle analysis found silencing of hsa_circ_0000725 could lead to the accumulation of cells in the G0/G1 stage, while the silencing of hsa_circ_0008826 shows no difference. **(C,D)** Apoptosis analysis found silencing of hsa_circ_0000725 could increase apoptosis, while the silencing of hsa_circ_0008826 shows no difference. **(E,F)** Invasion assay showed the silencing of hsa_circ_0000725 and hsa_circ_0008826 could reduce the invasion ability of CRC cells.

Flow cytometry assays found that the silencing of hsa_circ_0000725 could significantly lead to the accumulation in the G0/G1 stage of CRC cell, while the silencing of hsa_circ_0008826 showed a subtle change of cell cycle stages (Figures 5A, B). Meanwhile, apoptosis assays found that the silencing of hsa_circ_0000725 could promote apoptosis; however, the silencing of hsa_circ_0008826 made no difference in apoptosis (Figures 5C, D). The invasion assay showed that both hsa_circ_0000725 and hsa_circ_0008826 promoted the invasion ability of DLD-1 (Figures 5E, F).

## Discussion

As CRC has a high incidence and high mortality, early diagnosis and intervention methods are essential in the successful management of the disease. Thus, seeking efficient biomarkers for diagnosis or specific targets for treatment is an urgent priority. Numerous studies have found novel biomarkers and potential therapeutic targets for CRC (Mai et al., 2020; Feng et al., 2021; Ning et al., 2021; Sun et al., 2021; Wu et al., 2021b). However, few can be introduced truly in clinical use. Therefore, finding stable and detectable molecules that can be used in clinical applications is important. CircRNAs possess a unique circle structure that is stable and easily detected. In the past, they were not believed to have coding ability. However, in recent years, a gradual number of studies have discovered that circRNAs can be cap-independent in translation (Pamudurti et al., 2017), and the translation process can be conducted through other approaches, for example, IRES-mediated and m6A-mediated (Yang et al., 2017).

Before our study, emerging evidence had shown the importance of the coding function of circRNAs. In 2017, Legnini and his colleagues first identified that circ-ZNF609, which specifically controls myoblast proliferation, can be translated into a protein in myogenesis (Legnini et al., 2017). Another study illustrated the translational landscape of the human heart by analyzing the translatomes of 80 human hearts and also discovered the coding potential of circRNAs (van Heesch et al., 2019). Our study has constructed a strategy that can be used to identify circRNAs with coding potential. The coding potential of circRNAs has promising application prospects. Recently, a study used the coding potential of circRNA to produce a circRNA vaccine that encoded the trimeric RBD of the SARS-CoV-2 spike protein with great efficacy, high design flexibility, and fast manufacturing speed compared to the traditional methods of vaccine production (Qu et al., 2021). Peptides encoded by circRNAs also have important functions in other cancers. In CRC, it was reported that peptide circPPP1R12A-73aa encoded by hsa_circ_0000423 can promote tumor pathogenesis and metastasis of CRC through the Hippo pathway (Zheng et al., 2019), and circFNDC3B-218aa encoded by circFNDC3B can regulate Snail to inhibit tumor progression and EMT in CRC (Pan et al., 2020). Zhang and his colleagues found several circRNAs that could encode peptides and function significantly in glioma (Yang et al., 2018; Zhang et al., 2018; Xia et al., 2019; Gao et al., 2021; Wu et al., 2021b). The FBXW7-185aa encoded by circ-FBXW7 can inhibit the proliferation and cell cycle acceleration of glioma (Yang et al., 2018), and the circular SHPRH as well as circular AKT3 could encode tumor-suppressed peptides in glioblastoma (Zhang et al., 2018; Xia et al., 2019). Meanwhile, a unique 14-a.a. peptide encoded by circ-E-Cad can promote the tumorigenicity of glioblastoma by activating the EGFR-STAT3 signal (Gao et al., 2021). Moreover, the phenomenon of coding circRNAs has also been observed in liver cancer (Liang et al., 2019; Zheng et al., 2019). The Wnt pathway is a classical tumor suppressor pathway; studies have found that circβ-catenin could influence a wide variety of Wnt pathway-related genes and encode a novel 370-a.a. peptide. This peptide can help to stabilize full-length β-catenin by antagonizing GSK3β-induced β-catenin phosphorylation and degradation (Liang et al., 2019). Furthermore, a novel protein, MAPK1-109aa, encoded by circMAPK1, can inhibit the progression of gastric cancer by suppressing the activation of the MAPK pathway (Jiang et al., 2021). Growing evidence has demonstrated the importance of circRNA-derived peptides in tumorigenesis and tumor development, which may be a potential biomarker or therapeutic target for cancer treatment in the future. Our strategy may be a convenient approach for searching the coding potential of circRNAs not only in CRC but also in other cancers.

In this study, we constructed a strategy to efficiently search the coding potential circRNAs in CRC. In total, the candidate list lasts nine circRNAs, including hsa_circ_0000725, has_circ_0007429, hsa_circ_0008501, hsa_circ_0067080, hsa_circ_0005654, hsa_circ_0006088, hsa_circ_0007364, hsa_circ_0008199, and hsa_circ_0008826 (Figures 3A, B and Supplementary Figures S1 and S2). Owing to the unique sequence of the novel peptides, we focused on those that had sequences identifiable by antibodies in their linear mRNA; two circRNAs were selected. Hsa_circ_0000725 and hsa_circ_0008826 were predicted to encode a 306-a.a. peptide and 475-a.a. peptide, respectively. Western blot analysis showed that hsa_circ_0000725- and hsa_circ_0008826-derived peptides were expressed in multiple CRC cells, which is almost in accordance with the qPCR results. Then we preliminarily explore the function of hsa_circ_0000725 and hsa_circ_0008826; both of them could significantly promote the proliferation and colony formation as well as invasion ability of CRC. Besides, the silencing of hsa_circ_0000725 could lead to the accumulation of cells in the G0/G1 stage as well as the increase of apoptosis rate. In a word, these peptide-coding hsa_circ_0000725 and hsa_circ_0008826 could promote the proliferation as well as invasion ability of DLD-1; however, whether their coded peptides make the most contribution to this phenomenon still needs further validation.

Our findings helped to construct a new schedule to scan potential coding circRNAs, which may be very practical in functional studies on circRNAs. However, our study still had limitations and shortcomings. The function of the circRNA itself could not be excluded, including the competing endogenous RNA (ceRNA) mechanism or RNA-binding protein mechanism. Whether the circRNA-derived peptides functioned in a pivotal role still needs further validation; specific antibodies are needed to identify these peptides, and overexpression vectors are needed to clarify the findings. Therefore, with the limitations considered, our preliminary exploration indicated that hsa_circ_0000725 and hsa_circ_0008826 possessed the ability of coding peptides and could promote the proliferation and invasion of CRC cells, but only hsa_circ_0000725 could influence the cell cycle and apoptosis rate of colorectal cells. Our strategy for scanning potential coding circRNAs was found to be effective. And these potential coding circRNAs may be good choices when seeking CRC therapeutic targets or diagnosis biomarkers.

## Data Availability

Publicly available datasets were analyzed in this study. These data can be found here: The data that support the findings of this study are available from the corresponding author upon reasonable request.
