# Supplementary material for: Strategy for Scanning Peptide-Coding Circular RNAs in Colorectal Cancer Based on Bioinformatics Analysis and Experimental Assays
Source: Front Cell Dev Biol. 2022 Feb 25;9:815895. doi: 10.3389/fcell.2021.815895 (PMC8913576; doi:10.3389/fcell.2021.815895)
Supplement: Supplementary file 1 [file Table1.DOCX]

**Table S1. Primers in use.**

| **Name** | **Forward** | **Reverse** |
| --- | --- | --- |
| hsa_circ_0000725 | GCATCCGGTGTGACCAAAAG | AAAACAACTGGGTGGTCAGGG |
| hsa_circ_0008826 | GCCTGCAAGGGAAGCTTGT | ACTGCCTGTAGCACAACAGAT |
| has_circ_0007429 | GGGAACATGCACAGTGTCAAC | GGTCTGAACCTTCCAAGCCA |
| hsa_circ_0008501 | AACCCTGAGACAAGGTCCAC | CCCGGCTTCAGAAAGATGCT |
| hsa_circ_0067080 | CAGCCGTCTGCAAGGTCTC | TTTGCCCTCAGATCACACCG |
| hsa_circ_0005654 | TCACACCAGGGAGATCCTAAG | GGGTCGTTTTTCTGAGTGGGT |
| hsa_circ_0006088 | AGCGATTTCCGGATGAAACTG | TCTGTACTTCATGGGCGCTG |
| hsa_circ_0007364 | ATCCACGTTCTAGTTTTTCGTTGG | TCTCGGTGTCCAGGAGTCTT |
| hsa_circ_0008199 | CAAGACAAGAGAAACAGCACCC | TCCTGTTCCACTCGCAGTTC |
| GAPDH | ATGATTCCACCCATGGCAAATTC | TGGTTCACACCCATGACGAA |

**Table S2. Sequencing analysis information of 57 candidate circRNAs.**

| **circRNA ID** | **Host gene** | **Weight** | **Start Position** | **End Position** | **Protein Length** | **Unique sequence** |
| --- | --- | --- | --- | --- | --- | --- |
| hsa_circ_0000370 | FLI1 | 18.57 kDa | 352 | 1j*+41 | 165 aa | GGSVGGERRPVPL |
| hsa_circ_0000504 | TUBGCP3 | 27.09 kDa | 73 | 1j+29 | 235 aa | VFCSIRSNS |
| hsa_circ_0000725 | BANP | 33.52 kDa | 69 | 1j+26 | 306 aa | DQKPAPL |
| hsa_circ_0001119 | NDUFA10 | 13.57 kDa | 132 | 1j+24 | 114 aa | WTTTTR |
| hsa_circ_0001394 | TBC1D14 | 26.70 kDa | 18 | 1j+25 | 248 aa | SFSLDQDD |
| hsa_circ_0001727 | ZKSCAN1 | 23.78 kDa | 89 | 1j+41 | 206 aa | GIVKKHIIKPPRT |
| hsa_circ_0002884 | PICALM | 23.07 kDa | 612 | 2j+21 | 219 aa | HIKAAQ |
| hsa_circ_0003026 | USP10 | 22.07 kDa | 499 | 1j+50 | 217 aa | ASSIQWNSSVWHTGCG |
| hsa_circ_0003098 | BANP | 34.04 kDa | 62 | 1j+36 | 307 aa | RRPPDYSNTQQ |
| hsa_circ_0003250 | MRRF | 11.51 kDa | 90 | 1j+40 | 105 aa | HQRERTVPNQSEY |
| hsa_circ_0004853 | CD97 | 16.33 kDa | 36 | 1j+39 | 149 aa | APPWRASSPSRT |
| hsa_circ_0005927 | VDAC3 | 10.06 kDa | 140 | 1j+33 | 90 aa | EWEIEGLL |
| hsa_circ_0006088 | SPTAN1 | 19.20 kDa | 49 | 1j+49 | 167 aa | MKLIPRQPPRGSLLV |
| hsa_circ_0006174 | RAD23B | 9.57 kDa | 69 | 1j+16 | 98 aa | RQNPQ |
| hsa_circ_0006958 | ACSF3 | 35.02 kDa | 194 | 1j+129 | 316 aa | SHRPSGFQRQAWCLPQEDEHLHCLHLIVPFHLLKQLCLPLL |
| hsa_circ_0007015 | PTPN14 | 15.87 kDa | 155 | 1j+64 | 135 aa | SYLPEGHTGHGHPFPCLEEHR |
| hsa_circ_0007144 | PTPRM | 20.74 kDa | 321 | 1j+22 | 186 aa | SPRCCSH |
| hsa_circ_0007364 | PTP4A2 | 10.87 kDa | 594 | 1j+96 | 94 aa | FRWNIRCTFMAILSVRADFCQAQHSIFADK |
| hsa_circ_0007905 | STX6 | 16.15 kDa | 4 | 1j+47 | 144 aa | GHERSDVNFICAGIS |
| hsa_circ_0007983 | GABPB1 | 22.39 kDa | 121 | 1j+134 | 212 aa | AGNFSTSSSSTVWSLFHHRGTAASWCEQRCQNQSGPNTITYGSF |
| hsa_circ_0008199 | ATXN10 | 26.68 kDa | 419 | 1j+69 | 235 aa | RNSTQDYLPKSSGYPKEIFSCC |
| hsa_circ_0008812 | RAD23B | 18.12 kDa | 470 | 2j+26 | 176 aa | GESTERED |
| hsa_circ_0008826 | DHTKD1 | 53.48 kDa | 183 | 1j+111 | 475 aa | GSLWAVPSSMSMETAQRKWSVPHDWLLNTNASSARM |
| hsa_circ_0009006 | LDB2 | 11.38 kDa | 138 | 1j+57 | 99 aa | SAGPSSPVTLALCLKEG |
| hsa_circ_0012300 | PIK3R3 | 32.76 kDa | 27 | 1j+22 | 276 aa | SSTKAT |
| hsa_circ_0017726 | DHTKD1 | 51.86 kDa | 168 | 1j+27 | 458 aa | LIMALPGW |
| hsa_circ_0034762 | MAPKBP1 | 21.41 kDa | 131 | 1j+96 | 192 aa | CGCVVQSPETQTAPHPQQFQENHHCPCLLP |
| hsa_circ_0036408 | ETFA | 16.08 kDa | 407 | 2j+41 | 152 aa | LIATISEYPGNS |
| hsa_circ_0043278 | TADA2A | 8.52 kDa | 49 | 1j+23 | 74 aa | YFRFSCP |
| hsa_circ_0046430 | FOXK2 | 44.12 kDa | 149 | 1j+38 | 418 aa | GAHSGSRAQTSR |
| hsa_circ_0047814 | ZNF532 | 82.99 kDa | 18 | 1j+18 | 787 aa | NICSN |
| hsa_circ_0061774 | BACE2 | 23.15 kDa | 376 | 1j+17 | 210 aa | ATDSR |
| hsa_circ_0063305 | CSNK1E | 7.80 kDa | 96 | 1j+51 | 71 aa | VPTSPLVRKSPSSWSV |
| hsa_circ_0067080 | ITGB5 | 19.23 kDa | 255 | 1j+54 | 169 aa | VSTYALVEVPPHVKNVC |
| hsa_circ_0067434 | RYK | 17.95 kDa | 27 | 1j+24 | 156 aa | IKLLKFR |
| hsa_circ_0087641 | CDC14B | 43.27 kDa | 126 | 1j+73 | 376 aa | SPLFCHSLQQTKECIKCTLFQHR |
| hsa_circ_0088865 | SPTAN1 | 17.84 kDa | 13 | 1j+13 | 155 aa | LLV |
| hsa_circ_0001513 | LNPEP | 41.24 kDa | 24 | 1j+27 | 371 aa | IGFSSPGI |
| hsa_circ_0008501 | RERE | 15.86 kDa | 148 | 1j+124 | 136 aa | STTPRPVADLQLLLCVTPQHALCRWHHSHHSIFLKPGEGL |
| hsa_circ_0006123 | KCNH1 | 12.86 kDa | 145 | 1j+68 | 117 aa | WHQQPVQLSKSCPAAPSWASGP |
| hsa_circ_0006528 | PRELID2 | 12.12 kDa | 216 | 1j+25 | 101 aa | FPRFEKD |
| hsa_circ_0002702 | RUSC2 | 70.71 kDa | 93 | 1j+17 | 676 aa | AKLS |
| hsa_circ_0005654 | PRDM5 | 30.04 kDa | 111 | 1j+114 | 253 aa | ILRKSLYVQCAIKSVLQHQAYRNIERFMRYLIVKNV |
| hsa_circ_0042435 | SPECC1 | 19.75 kDa | 242 | 1j+12 | 178 aa | TCG |
| hsa_circ_0003273 | TRIP12 | 31.86 kDa | 50 | 1j+17 | 305 aa | MNGSV |
| hsa_circ_0000567 | SETD3 | 26.96 kDa | 9 | 1j+30 | 234 aa | SEKWVRRVE |
| hsa_circ_0087897 | TMEM245 | 76.77 kDa | 1995 | 2j+31 | 688 aa | SGRWWLAMC |
| hsa_circ_0029976 | NBEA | 48.08 kDa | 28 | 1j+31 | 426 aa | WLVENLTWR |
| hsa_circ_0002153 | MID2 | 30.76 kDa | 57 | 1j+54 | 269 aa | KAQPPWFLMPQEDYFH |
| hsa_circ_0000267 | FAM53B | 23.86 kDa | 234 | 1j+120 | 219 aa | KMTDGETWTGNALFRLTNRAPASGNACLKRTAHYGTGRQ |
| hsa_circ_0011950 | HIVEP3 | 188.39 kDa | 522 | 1j+13 | 1739 aa | RRA |
| hsa_circ_0009580 | RERE | 6.55 kDa | 125 | 1j+42 | 63 aa | EAEQYPGGDSCRS |
| hsa_circ_0009581 | RERE | 5.56 kDa | 230 | 1j+12 | 53 aa | REV |
| hsa_circ_0001573 | RREB1 | 18.15 kDa | 285 | 1j+88 | 170 aa | LLRLCVPG VVALRCDPAH VWAQPGSAPQ |
| hsa_circ_0007429 | RREB1 | 17.79 kDa | 166 | 1j+67 | 163 aa | SVNEYYQEK KTRGQHRLIF YSV |
| hsa_circ_0079958 | HECW1 | 26.92 kDa | 43 | 1j+26 | 233 aa | ECLPS LHQ |
| hsa_circ_0004349 | RAC1 | 8.80 kDa | 156 | 2j+12 | 78 aa | SCR |

*j: times of spanning junction site
