# Supplementary material for: Strategy for Scanning Peptide-Coding Circular RNAs in Colorectal Cancer Based on Bioinformatics Analysis and Experimental Assays
Source: Front Cell Dev Biol. 2022 Feb 25;9:815895. doi: 10.3389/fcell.2021.815895 (PMC8913576; doi:10.3389/fcell.2021.815895)

Supplementary Figure 1. The expression level of rest 7 circRNAs (A) and the identification of their unique a.a. sequence (B).

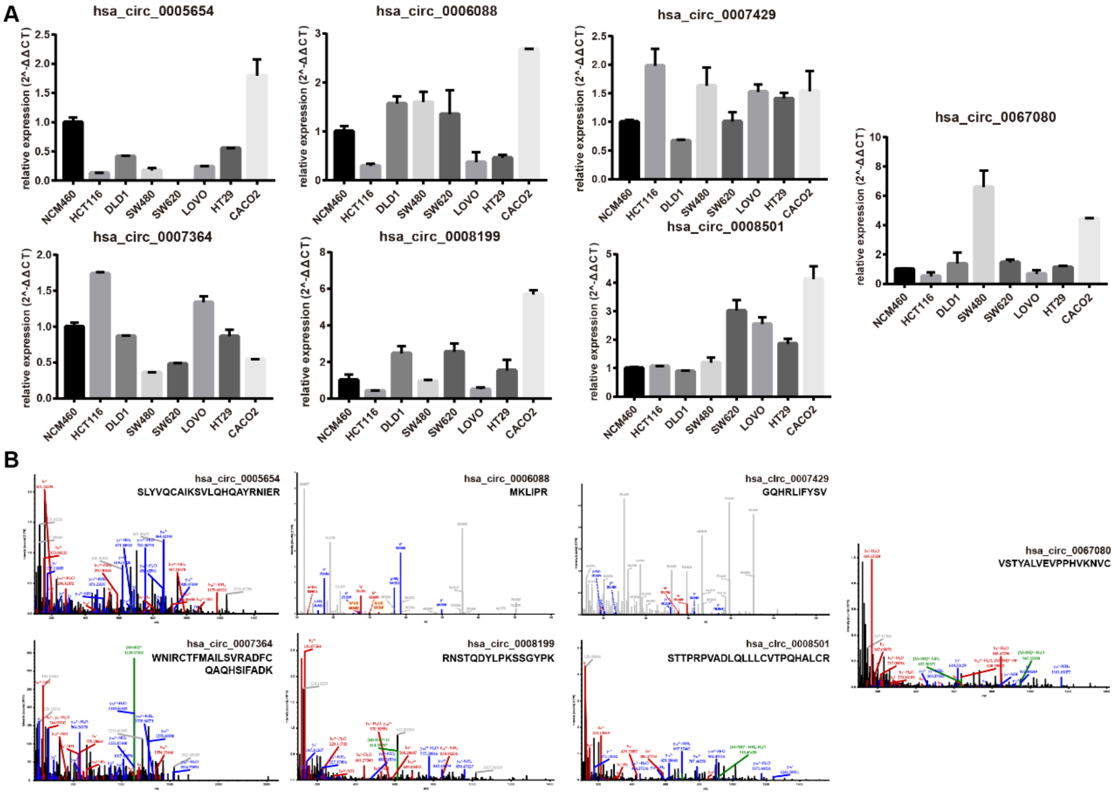

Supplementary Figure 2. The basic structures of rest 7 circRNAs.

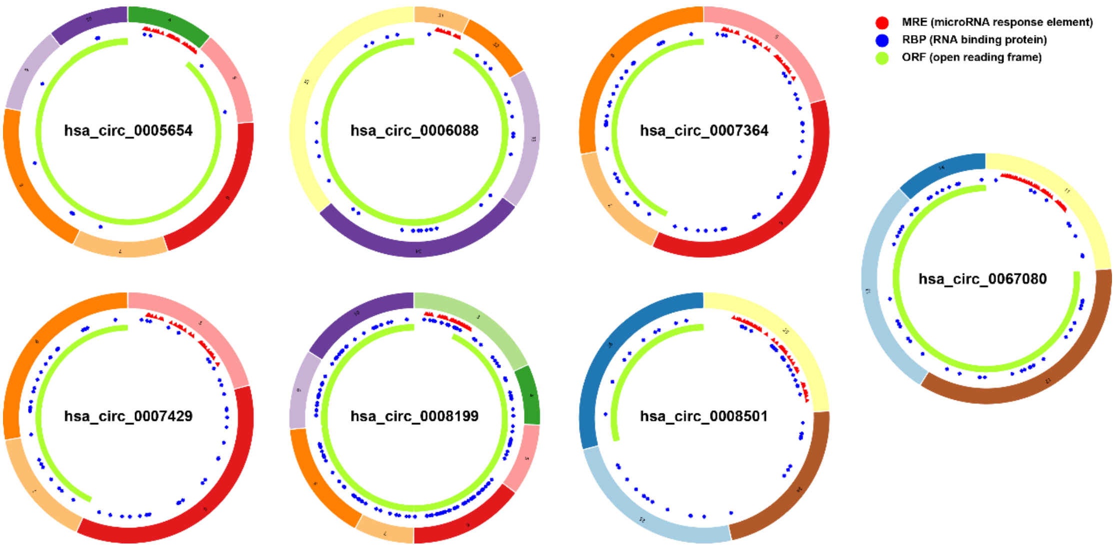

Supplement: Supplementary file 2 [file DataSheet1.PDF]
